# Sheep Reared on Sewage Sludge–Treated Pasture: Flawed Conclusions

**DOI:** 10.1289/ehp.114-a87

**Published:** 2006-02

**Authors:** Tim Evans

**Affiliations:** Tim Evans Environment, Ashtead, Surrey, England, E-mail: tim@timevansenvironment.com

I read the article by Paul et al. (2005) with interest. Although the authors devoted considerable energy and resources to their study, I believe that the experimental design is fundamentally flawed and the authors’ conclusions are not supported by the facts. The flaw in this grazing experiment is that the control treatment, a pasture not treated with sewage sludge, is not a valid control. A valid experimental control should be as close to identical to the treatment(s) as possible, except for the factor under investigation.

In the study of Paul et al. (2005), the pastures received 250 kg nitrogen/ha from sewage sludge or from mineral fertilizer. Under the climatic conditions of the experiment, the sludge nitrogen would be equivalent to about 70 kg nitrogen from mineral fertilizer, with much of the rest of the nitrogen (and carbon) going into soil organic matter stocks. Thus, the “control” received three times as much plant-available nitrogen as the sludge pasture, and the herbage yield would have been greater. The lower herbage yield and more restricted diet on the sludge plot is borne out by the lighter weights of the ewes and the fetuses.

In addition to the difference in nitrogen, there was almost certainly a difference in the phosphorus supply. Paul et al. (2005) did not describe fertilizer applications apart from nitrogen, but I doubt that they added as much phosphorus to the control plot as they did to the treated plot in the form of sludge content. There is also the question of the other nutrients that would have been added in the sludge (potassium, magnesium, sulfur, calcium, and minor nutrients).

The lesser amount of available nitrogen and the much greater phosphorus (and the other nutrients) over ≥ 7 years would have almost certainly changed the sward composition. For example, there would almost certainly be much more clover in the sludge plots. Clover and other legumes are rich in phytoestrogens; therefore, if the effects observed by Paul et al. (2005) were due to endocrine-active substances in the diet, these substances could well have been phytoestrogens.

Paul et al. (2005) noted that some authors have reported similar effects in sheep on restricted diets, but other authors have not found the effects; therefore, this appears inconclusive. Paul et al. (2005) found physiologic effects but did not prove causation.

From the results of Paul et al. (2005), one could conclude that mineral nitrogen increased the number of quadruplets (which is bad from a farmer’s point of view because the ewe does not have enough milk for four lambs) and sludge gave consistently more triplets (good for farmers), but that would not be accurate. Based on their data, one could also say that statistically significantly more ewes escape from mineral nitrogen–fertilized fields than from sludge-treated ones, but that would be silly.

The diets of the two populations were different because the pastures were managed differently and, as a consequence, the animals responded differently; it would not be valid to say more.
